# The p*K*_a_ Distribution of Drugs: Application to Drug Discovery

**Published:** 2007-09-17

**Authors:** David T. Manallack

**Affiliations:** Department of Medicinal Chemistry, Victorian College of Pharmacy, Monash University, 381 Royal Parade, Parkville, 3052, Australia.

**Keywords:** pK_a_, dissociation constant, distribution, drugs, absorption, ADME, bioavailability, drug discovery, pharmacokinetics, acids, bases, ampholytes

## Abstract

The acid-base dissociation constant (p*K*_a_) of a drug is a key physicochemical parameter influencing many biopharmaceutical characteristics. While this has been well established, the overall proportion of non-ionizable and ionizable compounds for drug-like substances is not well known. Even less well known is the overall distribution of acid and base p*K*_a_ values. The current study has reviewed the literature with regard to both the proportion of ionizable substances and p*K*_a_ distributions. Further to this a set of 582 drugs with associated p*K*_a_ data was thoroughly examined to provide a representative set of observations. This was further enhanced by delineating the compounds into CNS and non-CNS drugs to investigate where differences exist. Interestingly, the distribution of p*K*_a_ values for single acids differed remarkably between CNS and non-CNS substances with only one CNS compound having an acid p*K*_a_ below 6.1. The distribution of basic substances in the CNS set also showed a marked cut off with no compounds having a p*K*_a_ above 10.5.

The p*K*_a_ distributions of drugs are influenced by two main drivers. The first is related to the nature and frequency of occurrence of the functional groups that are commonly observed in pharmaceuticals and the typical range of p*K*_a_ values they span. The other factor concerns the biological targets these compounds are designed to hit. For example, many CNS targets are based on seven transmembrane G protein-coupled receptors (7TM GPCR) which have a key aspartic acid residue known to interact with most ligands. As a consequence, amines are mostly present in the ligands that target 7TM GPCR’s and this influences the p*K*_a_ profile of drugs containing basic groups. For larger screening collections of compounds, synthetic chemistry and the working practices of the chemists themselves can influence the proportion of ionizable compounds and consequent p*K*_a_ distributions. The findings from this study expand on current wisdom in p*K*_a_ research and have implications for discovery research with regard to the composition of corporate databases and collections of screening compounds. Rough guidelines have been suggested for the profile of compound collections and will evolve as this research area is expanded.

## Introduction

An awareness of the influence of the acid-base dissociation constant, p*K*_a_, on the biopharmaceutical properties of drugs and chemicals has long been established within the pharmaceutical and chemical industry. As the majority of drugs are weak acids and/or bases, knowledge of the dissociation constant in each case helps in understanding the ionic form a molecule will take across a range of pH values. This is particularly important in physiological systems where ionization state will affect the rate at which the compound is able to diffuse across membranes and obstacles such as the blood-brain barrier (BBB). The p*K*_a_ of a drug influences lipophilicity, solubility, protein binding and permeability which in turn directly affects pharmacokinetic (PK) characteristics such as absorption, distribution, metabolism and excretion (ADME)^1^–^5^. The well established association between p*K*_a_ and PK has also resulted in the requirement for p*K*_a_ values to be measured for regulatory compliance (e.g. FDA^6^). Formulation procedures for optimizing drug delivery also benefit from the determination of the p*K*_a_. Given the importance of this parameter to the drug industry^7^, it follows that an ability to estimate or measure^8^ the p*K*_a_, together with a knowledge of their distribution, will be of great benefit. This is particularly important when contemplating the large number of compounds that can be considered for screening purposes (e.g. combinatorial libraries, third party compound collections). Ideally, these sets of compounds should be representative of drug-like substances as a whole with regard to the proportion of ionizables and the distribution of the p*K*_a_ values themselves.

An estimate of likely ADME characteristics can be obtained using p*K*_a_ values and various other properties such as molecular weight (MW), partition coefficient (logP), number of hydrogen bond donors (hdon) and acceptors (hacc), and polar surface area (PSA)^9^. The p*K*_a_ values themselves represent useful pieces of physicochemical information but in isolation they have limited value. From the perspective of designing combinatorial libraries or buying sets of compounds from third party suppliers then it is important to know what the overall profile of a collection should resemble with regard to a range of physicochemical properties. Therefore, in order to complement properties such as MW, logP, hdon, hacc and PSA, information regarding the proportions of acids and bases, and the distribution of p*K*_a_ values is required. In medicinal chemistry there are many instances where research is influenced by rules of thumb. This could be described as a collective wisdom amongst the medicinal chemistry community where the ‘rules’ have not been fully researched or described. Such might have been the case with the Lipinski study^10^ where some of the underlying principles were roughly known and applied prior to their publication. Certainly for p*K*_a_ distributions, these have not been fully documented in the literature. It is on this basis that the current study has sought to explore the proportions of acids and bases and to detail the distribution of p*K*_a_ values for a set of general drug-like molecules.

### Drug-likeness

In recent years there have been numerous studies exploring methods to improve the efficiency of the early stages of new medicines research. The aim of all these studies has been to reduce the development time from the initiation of a project through to the selection of a clinical candidate. Much of it has focused on the ‘drug-like’ or ‘lead-like’ nature of screening compounds or synthetic candidates^10^–^14^. The argument raised was that if compounds were selected for optimization that required a considerable number of synthetic cycles to produce novel analogues that address ADMET (T = toxicity) deficiencies then this lengthened the time needed to arrive at a clinical candidate. If, however, the compound was ‘drug-like’, or perhaps more preferably ‘lead-like’^15^ from the outset, then it should be easier to arrive at the appropriate biopharmaceutical properties and in a shorter timeframe^16^. Such aspirations are based on sound logic and have been implemented within the current practices of the pharmaceutical industry^10^,^17^. One of the simplest of these procedures is a structure and functional group filter that removes compounds considered unsuitable as hits such as those containing toxic functional groups^18^.

Research into drug-like and lead-like concepts has explored a range of ideas looking at structural characteristics and physicochemical properties. These studies have included examinations of molecular frameworks^19^,^20^, molecular properties^12^–^14^, ^21^, ^22^ and the prediction of ADME parameters^23^ to name but a few. In addition, compounds that target the CNS have also been analyzed to profile their physicochemical characteristics and to predict CNS activity^24^–^26^. As such, it is becoming entrenched within the medicinal chemistry community to look extremely closely at the characteristics of the molecules they deal with and to work on those known to have suitable properties. Once again, it makes logical sense to operate most of your time in areas where there is a history of successful outcomes and where efficiencies can be garnered.

**Figure 1.**
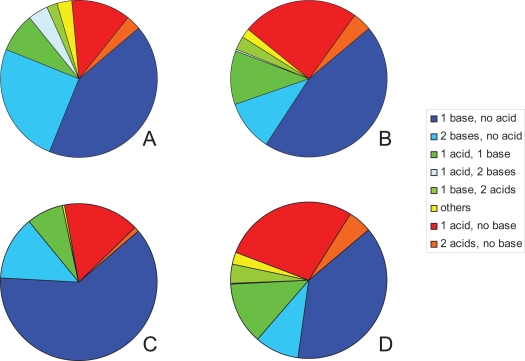
Pie charts showing the distribution of acids and bases from the findings of Comer and Tam^29^ outlining the survey conducted by Tim Mitchell^30^ on the acid base distribution using the 1999 WDI database, (**A**); the results from the 582 Williams compound dataset^33^, (**B**); the 174 CNS compound subset, (**C**); and the 408 non-CNS compound subset, (**D**). The data associated with these diagrams is given in Tables 1 and 2.

Our knowledge of the overall proportion of acids, bases and p*K*_a_ distributions is less understood than other aspects of drug and lead-likeness. For example, statements often describe drugs as ‘typically weak acids and/or weak bases’. The proportion of drugs with an ionizable group has been estimated at 95%^27^ while an analysis of the 1999 *World Drug Index* (WDI^28^) showed that only 62.9% of that collection were ionizable between a pH of 2 and 12^29^, ^30^. Wells also estimated that 75% of drugs are weak bases, 20% weak acids and the remainder contained non-ionics, ampholytes and alcohols^27^. A breakdown of the WDI set of ionizable compounds showed that two thirds of them had either a single basic group or two basic groups (Figure 1A). The next major group of compounds containing one or two acids made up 14.6% of this set while simple ampholytes with one acid and one base comprised 7.5%. To analyze the WDI database (51,596 compounds) the Chem-X software^31^ was used to discriminate acids and bases. The details of which functional groups were used is not easily discernable, however the concept of exploring a p*K*_a_ range of 2–12 is admirable given that the term ionizable used by Wells may possibly have encompassed a greater proportion of compounds. This may also suggest why only 62.9% of compounds in the WDI were considered of interest compared to Wells figure of 95%^27^. It should be noted (and presumed) that the two sets of drugs considered by the individual authors would have differed. On a smaller set of compounds (*n* = 53) with known capacity to cross or not cross the BBB it was^32^ concluded that “compounds with minimally one charge with a p*K*_a_ <4 for acids and correspondingly a p*K*_a_ >10 for bases do not cross the BBB by passive diffusion.” The references cited above are among the few that touch on both p*K*_a_ and the proportion of ionizable compounds within a set of drugs. It may be that dealing with p*K*_a_ is occasionally troublesome for a number of reasons, e.g. access to measured data is not simple, calculation of large numbers of p*K*_a_ values is cumbersome and compounds may contain variable numbers of ionizable groups. Consequently the p*K*_a_ does not lend itself to simple calculation and comparison, such as molecular weight or polar surface area (PSA) might allow.

### p*K*_a_ data sources and analysis

In order to conduct an analysis of the proportion of acids and bases, and p*K*_a_ distributions, suitable databases of p*K*_a_ values are required. Several sets of p*K*_a_ values are available such as PhysProp (Syracuse Research Corporation, North Syracuse, USA), Williams compilation in Foye’s textbook^33^, the Merck Index^34^, Avdeef 35, IUPAC and related compilations^36^–^41^, CRC Handbook^42^, Lange’s Handbook of Chemistry^43^, ACD/labs software and database^44^ as well as the general literature. In some cases these data resources do not assign p*K*_a_ values to particular functional groups. The Williams set (see Methods for details)^33^ used in this current study simply specifies whether the p*K*_a_ value is derived from an acid or a base and this feature was an important factor in selecting this dataset for the analysis. Other issues to keep in mind are data quality as these compilations stem from many laboratories. In an ideal world it would be prudent to return to the original study to investigate how the measurements were undertaken and how problems (e.g. apparent p*K*_a_ values, decomposition, precipitation, poor UV absorbance, use of co-solvents, complex multi functional compounds) were handled. This perhaps is another reason why the p*K*_a_ distribution of drugs has not been described in detail for the analysis of drug-like character.

The goal of these analyses is to provide an indication of the spectrum of p*K*_a_ values and the proportion of acids and bases within a drug discovery environment. This is with particular regard to drugs that have made it to the marketplace so that this may influence drug discovery processes in general. It could be envisaged that analyses of corporate collections, third party suppliers and combinatorial libraries (real or virtual) are undertaken to determine whether their distributions match that of marketed drugs. Following this, decisions could be made to add to collections where certain classes of compounds or p*K*_a_ ranges are underrepresented and to influence synthetic directions. In the simplest sense it may add information regarding the overall composition of compound collections which can be discussed accordingly. Computational tools oriented to looking at ionizable groups as well as tautomer states^45^ have recently been established. One example is the ProtoPlex module within Sybyl^46^ which can populate a database with alternative tautomers and protomers for each compound. Other workers have also striven to represent compounds in the most appropriate way by considering ionizable groups and tautomers. Kenny and Sadowski^47^ described their technique which is able to apply formal charges to selected functional groups. They also emphasized the importance of their work in procedures such as virtual screening. Pospisil and co-workers also showed that tautomer state affected docking scores in virtual screening^45^ thus emphasizing the importance of considering p*K*_a_ on how we conduct drug discovery. Overall it is clear that the p*K*_a_ value(s) of a substance is fundamental to many areas of early and late stage discovery and that knowledge of p*K*_a_ distributions will be similarly important to improve how we discover and develop new medicines.

## Methods

To explore the proportion of ionizable compounds to non-ionizable compounds the World Health Organization’s (WHO) essential medicines list was employed (March 2005^48^). This represents a list of “minimum medicine needs for a basic health care system”, together with a set of complimentary medicines for priority diseases. It may be viewed as a mini-pharmacopoeia, however the makeup of the set will differ somewhat to more extensive lists of drugs. Nevertheless it serves to encompass a range of drug classes for a wide range of medical needs. Compounds were classified into three groups: those with an ionizable group within the p*K*_a_ range of 2–12 (determined using the ACD/labs software^44^), those without an ionizable group and a miscellaneous set containing proteins, salts and others (e.g. gases, mixtures, polymers, metal complexes, etc). The proportion of ionizable compounds was determined for the entire set and a selected subset that excluded the miscellaneous set.

The list of p*K*_a_ values compiled by Williams^33^ was used as the source of data for the present study. An examination of the list was undertaken and the original set of 599 compounds was reduced to a final set of 582 for analysis. Within this list the source references are given and most of the values come from Hansch in Comprehensive Medicinal Chemistry Volume 6^49^ which is itself a secondary literature compilation. The Williams list^33^ was chosen for its assignment of acids and bases, accessibility and representation of a range of compound classes. The initial curation step included removing duplicates (e.g. bupivacaine and levobupivacaine; where the p*K*_a_ is equivalent) and those compounds without a p*K*_a_ value. For inclusion the compound was required to have a clinical use (either past or current use) or was considered safe for human consumption or represented an interesting chemo-type (e.g. saccharin). Data misplaced in columns was adjusted and where p*K*_a_ values for acid and base groups had been swapped this was amended. In some cases incorrect values were revised (e.g. tiaprofenic acid) and compounds with non-standard names were excluded where this led to ambiguity of the correct substance.

In addition to this examination, an assessment was made regarding whether the compound was intended for CNS use. In some cases this was not easy to define particularly when the drug has been targeted towards peripheral sites but has CNS side effects. A classic example is the first generation of histamine H1 receptor antagonists that were developed for the treatment of hay fever but often caused drowsiness. Where sedative activity was listed as an indication for the drug then it was annotated as a CNS drug (e.g. trimeprazine). Cocaine, albeit used clinically as a local anaesthetic, has well known CNS effects and was also classified as a CNS substance. In some cases the classification was difficult to assign and, for the most part, the decision was based on the intended uses of the drug.

Analysis of the distribution of p*K*_a_ values was applied to three groups of compounds: those containing a single acid, a single base and ampholytes with 1 acid and 1 base. Histograms for the distributions required binning the compounds into ranges (i.e. 0.5 < X ≤ 1.5, 1.5 < X ≤ 2.5, etc). In each case column heights were expressed as a percentage. Ampholytes (1 acid, 1 base) were also further classified as either ordinary (base p*K*_a_ < acid p*K*_a_) or zwitterionic (base p*K*_a_ >acid p*K*_a_) compounds. In order to plot and compare the ampholytes the isoelectric point was determined ([acid p*K*_a_ + base p*K*_a_)/2] and the values binned in a similar manner to the p*K*_a_ values.

## Results

### (a) Acid and base proportions

The proportion of acids and bases in the Williams^33^ dataset of 582 compounds was determined by reviewing the p*K*_a_ data and summing the number of compounds containing a single base, single acid, and so forth. Table 1 (*Entire dataset*) shows that almost half the compounds had a single base (45.4%) while single acid compounds made up about a quarter of the total (24.4%). Ampholytes comprised 14.8% of the total of which 65 compounds (11.2%) were considered to be simple ampholytes containing a single acid and base. The other major group was those compounds with two basic groups representing 10.5% of the total. Figure 1B clearly shows the distribution of the 582 compounds demonstrating that over half the compounds are basic in nature (56.5%) [i.e. containing 1, 2 or 3 basic groups without an acidic group].

Splitting the entire list into CNS (*n* = 174) and non-CNS (*n* = 408) compounds allowed the construction of pie charts for each of these individual groups. Figure 1C, together with Table 1 (*CNS subset*) show that the CNS class of compounds is dominated by those containing a single basic group (62.1%). If these compounds are combined with those possessing 2 bases this represents 75.3% of the total. The proportion of compounds containing a single acid was 15.5% while ampholytes (13 compounds) only made up 7.5% of this subset.

The non-CNS group of compounds showed a distribution similar to the entire dataset of 582 compounds and this no doubt was influenced by the large number of compounds that make up this set (*n* = 408). Figure 1D and Table 1 (*Non-CNS*) demonstrate that compounds with one or two basic groups now comprise less than half the total (47.5%). The single acids comprised 28.2% and if combined with compounds containing two and three acids these make up about one third of the total. Simple ampholytes on the other hand made up 12.7% of this subset consisting of 52 compounds. Table 2 compares the percentage of compounds containing acids and bases between the Williams lists^33^ and the analysis conducted on the WDI^29^,^30^. In general the WDI has fewer compounds containing a single acid and a greater number of compounds with two basic groups. The number of compounds with a single basic group was similar between the entire Williams list and the WDI.

The Williams^33^ compilation did not, of course, list non-ionizable compounds as its prime interest was in those substances with a p*K*_a_ value. To estimate the proportion of non-ionizable compounds in a similar manner to the analysis by Comer and Tam^29^,^30^ the WHO essential medicines list was used as a minimum set of therapeutic substances and compounds. The WHO list was consolidated to 301 compounds from their March 2005 edition. Of these, 196 (65.1%) contained an ionizable group with a p*K*_a_ in the range 2–12. This result is very similar to that obtained by Mitchell of 62.9%^29^,^30^. If we remove the miscellaneous compounds (e.g. proteins, salts, mixtures, polymers, gases, etc) from the analysis then we obtain a figure of 77.5% of compounds that contain a relevant ionizable group. This is in contrast to the 95% estimate of Wells^27^ and may be a consequence of the small size of the WHO dataset and the inherent limitations for compounds to be included in the list. Alternatively, Wells^27^ may have included compounds with ionizable groups outside the p*K*_a_ range of 2–12.

### (b) p*K*_a_ Distribution of single acid containing compounds

**Figure 2.**
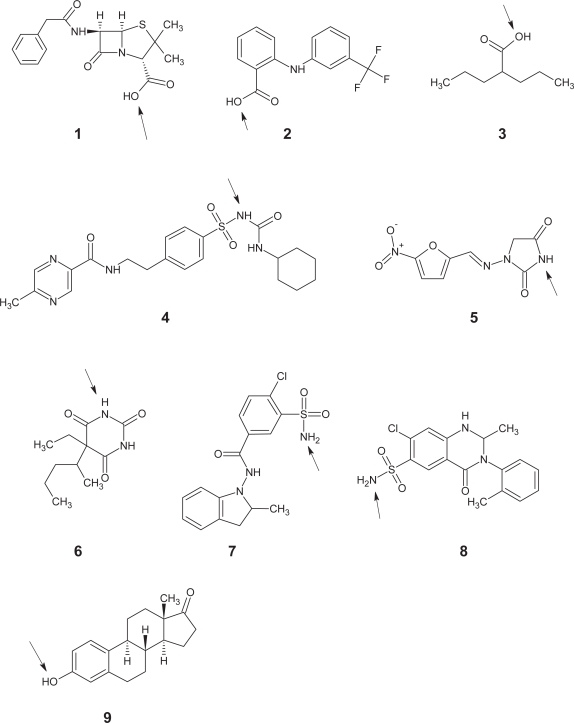
Chart showing nine acids with a range of p*K*_a_ values. In each case the acidic group has been highlighted with an arrow. Penicillin G (**1**, p*K*_a_ = 2.8), Flufenamic acid (**2**, p*K*_a_ = 3.9), Valproic acid (**3**, p*K*_a_ = 4.8), Glipizide (**4**, p*K*_a_ = 5.9), Nitrofurantoin (**5**, p*K*_a_ = 7.1), Pentobarbital (**6**, p*K*_a_ = 8.1), Indapamide (**7**, p*K*_a_ = 8.8), Metolazone (**8**, p*K*_a_ = 9.7), Estrone (**9**, p*K*_a_ = 10.8).

**Figure 3.**
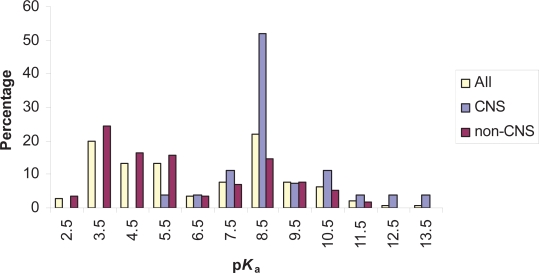
Histogram showing the p*K*_a_ distribution of compounds containing a single acidic group. Each group of columns contains a comparison of the entire set of single acids and those from the CNS and non-CNS subsets. Compounds were binned into 1 log unit ranges. For example, the column listed above 2.5 represents compounds with a p*K*_a_ greater than 1.5 and less than or equal to 2.5.

From the Williams set^33^ single acid containing compounds consisted of 142 substances and a representative sample of these is shown in Figure 2. The distribution of p*K*_a_ values is shown in Figure 3 and this also illustrates both the CNS and non-CNS classes. Each column is given as a percentage to allow for the differing sizes of each group. An examination of all 142 acids shows that there is a bimodal distribution with a dip in numbers at a p*K*_a_ of around 7.0. Compounds at the lower end of the scale largely contain carboxylic acids while those peaking around a p*K*_a_ value of 8.0 contained a large proportion of barbiturates.

Within the CNS class only 27 compounds had a single acid. While this is a low number, the distribution of p*K*_a_ values was nonetheless very interesting. Figure 3 shows that the majority of acids had a p*K*_a_ above 7 and only one fell below 6.1 (valproic acid = 4.8).

When the non-CNS class was inspected the bimodal distribution of p*K*_a_ values was again portrayed showing the dip in frequency close to 7.0. Within this set of 115 compounds those with lower p*K*_a_ values were predominantly carboxylic acids.

### (c) p*K*_a_ Distribution of single base containing compounds

**Figure 4.**
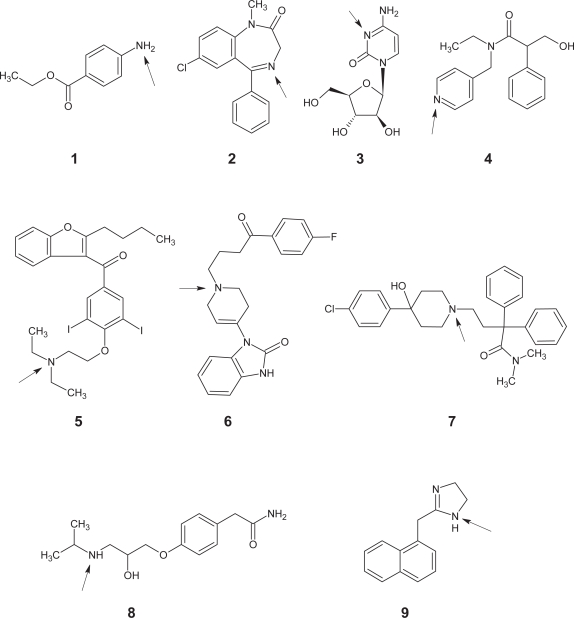
Chart showing nine bases with a range of p*K*_a_ values. In each case the basic group has been highlighted with an arrow. Benzocaine (**1**, p*K*_a_ = 2.5), Diazepam (**2**, p*K*_a_ = 3.4), Cytarabine (**3**, p*K*_a_ = 4.3), Tropicamide (**4**, p*K*_a_ = 5.3), Amiodarone (**5**, p*K*_a_ = 6.6), Droperidol (**6**, p*K*_a_ = 7.6), Loperamide (**7**, p*K*_a_ = 8.6), Atenolol (**8**, p*K*_a_ = 9.6), Naphazoline (**9**, p*K*_a_ = 10.9).

**Figure 5.**
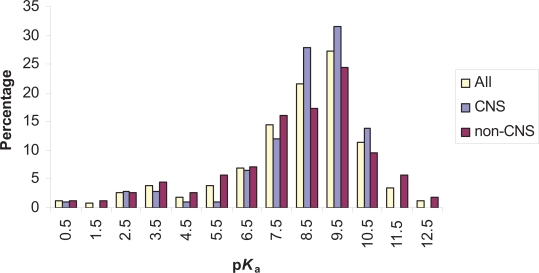
Diagram showing the p*K*_a_ distribution of compounds containing a single basic group. Each group of columns contains a comparison of the entire set of single bases and those from the CNS and non-CNS subsets. Compounds were binned into 1 log unit ranges as per Figure 3.

In contrast to the distribution of acids and perhaps as expected, the base p*K*_a_ values peaked at a value of 9.0. The majority of compounds had a p*K*_a_ value above 6.5 and these compounds typically contained a basic amine group. At the lower end of the p*K*_a_ scale various functional groups were represented (e.g. nitrogen containing heterocycles). Figure 4 shows a set of representative bases containing various heterocycles and amines. In all, 264 compounds contained a single base making up just under half of the total set analyzed. Figure 5 shows the distribution of base p*K*_a_ values ranging in value from 0.1 to 12.3. Once again the CNS and non-CNS classes have been included to allow a comparison of the three groups.

The CNS class (*n* = 108) showed a clear cut off at the high end of the p*K*_a_ scale. Indeed, there were no bases with a value above 10.5. Once again the majority of compounds had a p*K*_a_ above 7 and mostly consisted of amines. The distribution for the non-CNS class closely matched the overall pattern found for the entire dataset with a peak in p*K*_a_ values at around 9.0. p*K*_a_ values for the non-CNS compound set (*n* = 156) ranged from 0.3 to 12.3.

### (d) p*K*_a_ Distribution of simple ampholytes

**Figure 6.**
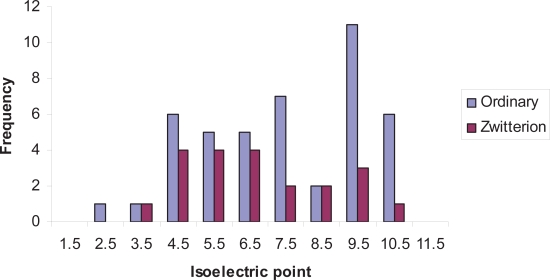
Histogram comparing the isoelectric points of both ordinary and zwitterionic ampholytes. In this case the frequencies of the distributions were shown to reflect the differing number of ordinary ampholytes (44 compounds) and zwitterionic ampholytes (21 compounds). Compounds were binned into 1 log unit ranges as per Figure 3.

In order to analyze the distribution of simple ampholytes (i.e. single acid and base) they were first classified as either ordinary or zwitterionic ampholytes and the isoelectric points were calculated. Figure 6 illustrates the range of isoelectric points for both the ordinary and zwitterionic ampholytes. While no clear pattern emerges this may be a reflection of the limited number of compounds (65) available for this analysis. The larger number of ordinary ampholytes at the high end of the scale represent simple phenols with alkylamine side chains (e.g. phenylephrine). If these compounds are left aside, those that remain tend to have isoelectric points between 3.5 and 7.5.

When the CNS and non-CNS drugs were compared interesting differences were observed. For the CNS class there were 13 simple ampholytes which made up only 7.5% of the 174 CNS compound subset. Of these 13 compounds there were six opioids and six benzodiazepines all of which were ordinary ampholytes. In contrast, the non-CNS subset contained 52 ampholytes comprising 20 zwitterions and 32 ordinary ampholytes. No doubt the predominance of ordinary ampholytes in the CNS class reflects the neutral character of these compounds at their isoelectric point where neutrality would favour CNS penetration.

## Discussion

### Overview of findings

One concern over the analyses conducted in this study may be the choice of datasets used. This is a problem that plagues any analysis of drug sets that aim to tease out trends in physicochemical characteristics. The set employed should of course be representative of drugs as a whole to enable reasonable conclusions to be drawn. To look at the proportion of ionizables the WHO essential medicines list^48^ was used which represents a small pharmacopoeia for priority health care needs. It is overrepresented in certain drug classes (e.g. antibiotics) and lacks a range of medicines which are costly or merely enhance the quality of life (e.g. selective serotonin reuptake inhibitors, HMG-CoA reductase inhibitors, PDE 5 inhibitors, etc.). Nevertheless it is a well thought-out list covering the majority of therapeutic classes. In contrast, the WDI dataset used by Comer and Tam^29^, ^30^ consisted of 51,596 compounds and could be viewed perhaps as a master list of drugs. The WDI, however, includes pesticides, herbicides and compounds that did not reach the market place. Given our desire to be representative of drugs it is not an ideal set and may be considered too encompassing. Our analysis therefore of the proportion of compounds that are ionizable is very dependent on the dataset used and provides results specific to that set. Another option is to examine all the drugs used commercially around the world such as those listed in Martindale^50^. This contains over 5000 drug monographs and an analysis based on this set would be an onerous task. The obvious alternative is to choose a smaller set that has undergone an evolutionary process to select useful therapeutic substances (e.g. through evidence-based therapy), such as the AHFS Drug Handbook^51^ (a subject of future research in this laboratory). Until such time that an agreed set of compounds can be selected to determine how many are ionizable the numbers generated here using the WHO list (65.1%) is comparable to the WDI findings of Comer and Tam^29^,^30^ (62.9%) and is far less than the 95% estimate described by Wells^27^. It is not clear which compounds Wells considered or how an ‘ionizable compound’ was defined. A more interesting analysis might be where strict criteria are used for compounds to be included in a survey. For example, organic compounds of molecular weight <1000 together with a use in mammalian therapy in an oral (or injected) form. For small organic substances this would give a better indication of the proportion of compounds possessing an ionizable group.

The Williams list of compounds^33^ could also be scrutinized in the same manner as the WHO essential medicines list. It is however, an extensive set of substances and represents a wide range of therapeutic classes. Once again better and more recent sets could be devised for this study and the Williams set was selected as a useful representative set and for the large number of compounds it contained. As mentioned above this aspect of the study is being addressed in future work in these laboratories using the compounds listed in the AHFS Drug Handbook^51^.

Until such time that these larger and more recent data sets are analyzed this present study provides an interesting insight into both the proportion of ionizable substances and the distribution of p*K*_a_ values. The catch all phrase describing drugs as mainly ‘typically weak acids and/or weak bases’ certainly holds true when the p*K*_a_ distributions are viewed (Figures 3 and 5). The power of the present analysis is to flesh out the bones to this simplistic description and provides a starting point for discussing p*K*_a_ distributions. In particular, the apparent biphasic distribution of acid p*K*_a_ values needs to be investigated further. Another important aspect to this research has been the scrutiny applied to CNS compounds. While, there is a general understanding concerning the principles behind the distribution of acid and base p*K*_a_ values for CNS drugs, this has not been well documented or presented in the literature. For example, it is known about the paucity of CNS compounds with acid p*K*_a_ values below 4.0 and base p*K*_a_ values above 10.0^32^. Also recognized is the sensibility of these values as charged substances do not easily cross the BBB. Acids with p*K*_a_ values below 4 will be in a charged state over 99% of the time at physiological pH as will bases with a p*K*_a_ above 10. The cutoff values described by Fischer and coworkers^32^ concur with the observations presented here, although only one compound had an acid p*K*_a_ below 6.1. The important aspect of this present study was to outline the distributions themselves to demonstrate the spectrum of p*K*_a_ values. Indeed, the overall implication is that this is valuable information when contemplating the properties needed for a drug or sets of screening compounds.

### Application of findings

The utility of the distributions described here may be applied to third party supplier databases for purchasing decisions regarding screening compounds. Either the ratio of ionizable to neutral compounds could be applied or the p*K*_a_ distributions could be used in the selection process. One thing that needs to be borne in mind is that the work described in this study has emerged from an analysis of drugs. Given that current screening efforts are oriented to lead-like molecules^15^ then the distributions need to be considered in this light. Certainly an analysis of an ideal screening set of lead-like compounds would yield the appropriate data. In the absence of this we need to look at the guidelines suggested for lead-like character. These follow the criteria outlined here: MW < 350, logP < 3 and affinity approximately 100 nM^16^. In other words there is scope for chemists to take a small molecule with reasonable activity and enter this into rounds of optimization for activity, selectivity and biopharmaceutical properties. The physicochemical criteria listed above are very simple, however p*K*_a_ and logD are not considered. Perhaps a simple ratio of ionizable to non-ionizable compounds needs to be suggested (e.g. 3:1, respectively). Furthermore the makeup of the ionizables also needs to be considered by selecting compounds with single acids, single bases and ampholytes, in approximately the ratios outlined in Table 2. More complicated combinations of acids and bases or those with 2 or more acids and bases should be kept to a minimum. These suggestions are purely speculative and are open to debate; suffice to say that the compounds should contain a mix of neutral and ionizables in roughly the ratios seen for drugs as well as allowing chemists the possibility of adding further ionizable groups to enhance activity and biopharmaceutical characteristics as part of the optimization process.

### Perspectives and future directions

Ionizable groups on drug molecules have two principal functions. The first is to modify overall polarity, which in turn controls other physicochemical properties, such as aqueous solubility or hydrophilicity. The second is to provide functional groups that can interact with target macromolecules in specific ways. Organic chemists, on the other hand, do not necessarily consider ionizable groups as first priority groups to include on a novel compound. A chemist, for ease of synthesis may prefer to work with non-polar compounds that are soluble in organic solvents. Another human consideration is the simplicity of the chemistry. Straightforward synthetic schemes will no doubt predominate to reduce the number of steps required. Given that ionizable groups often require protection means that additional synthetic steps are needed and introduces a further level of difficulty. Taking all this together suggests that organic compounds made to date will largely be lacking in ionizable groups. Furthermore, many of the third party suppliers need a large number of new substances for their catalogues which means that a high throughput is required from their chemists. High throughput will be a driver for simpler chemistry and, using the argument above, will result in compounds lacking ionizable groups. Of course, this trend has been identified and is being specifically addressed for compounds with utility in medicinal chemistry. This refers to Lipinski’s^10^ observations but the historic collections available will certainly be influenced by the (Darwinian) principle of ‘simple chemistry wins’.

Medicinal chemists also follow the principles of organic chemistry and prefer to introduce polar (ionizable) groups in the latter stages of a synthesis (e.g. protecting group removal). The last step of a synthesis can also be engineered to be one that can introduce diversity to generate a set of analogues. Third party screening compound suppliers, however, obtain a proportion of their catalogue from organic chemists rather than medicinal chemists. As such it may be that these offerings do not follow the same acid/base/p*K*_a_ distributions as drugs. Consequently, an examination of acid/base/p*K*_a_ distributions will be beneficial to ensure that a suitable mix of compounds is chosen for screening, irrespective of the source.

An overriding question fundamental to this study concerns the p*K*_a_ distributions themselves. Two separate influences will ultimately shape these findings. The first is chemical in nature concerning the functional groups that comprise the acid and base moieties. If we took the universe of organic compounds (a good representative subset might be the organic compounds contained in the CAS collection) and produced p*K*_a_ distribution plots then it would be possible to see how drugs compare. It may be that single acid containing compounds don’t exhibit a bimodal distribution and that drugs specifically lack groups with p*K*_a_ values around 7.0. Similar arguments could be directed at basic compounds and that the distributions we observe for drugs are a function of the regularly seen groups used in these compounds. Certainly, toxic functional groups will be very limited in the Williams set^33^ and this may also affect the p*K*_a_ distribution. The second driver for the p*K*_a_ distributions is biological in nature and is affected by membrane properties and the drug targets themselves. It is known that 7-transmembrane G-protein coupled receptors (7TM GPCR’s) have a key aspartic acid residue to recognize the amine group on their endogenous ligands^52^. The need for an amine in drugs that interact with 7TM GPCR’s is almost an absolute requirement. If we combine this with the fact that a high percentage of drug targets are 7TM GPCR’s^53^ then it will follow that amines will be well represented (particularly for CNS compounds) in the Williams set^33^. Our knowledge of p*K*_a_ distributions for a number of functional groups is quite reasonable but not when these are considered collectively. Presumably the pK_a_ value is a quantity which does not have a smoothly distributed continuum of values, but is necessarily multimodal because of the types of functional groups that exist in organic chemistry. In that sense, it is unlike logP, which has a much more broadly distributed set of values. This is a research area that will no doubt develop as larger populations of compounds are studied.

The task of identifying acids and bases in a database is a readily achievable task. A more difficult procedure is to estimate the p*K*_a_ values for these compounds. With regard to accuracy we preferably seek to predict within one log unit of the measured value. A variety of computational approaches are available and this topic was reviewed recently by Wan and Ulander^7^. A number of methods are used within the commercial packages (e.g. ACD/Labs^44^) such as the use of QSAR models based on Hammett analyses. Typically, a molecule is fragmented and the p*K*_a_ of the functional group is estimated by referring to a database of values with associated QSAR equations. Artificial neural network methods have also been used to estimate p*K*_a_ and the software available from Simulations Plus is one such example^54^. The ADME Boxes package from Pharma Algorithms^55^ also estimates the total number of ionizable groups and predicts the principle p*K*_a_ values. The other primary method of estimating p*K*_a_ values is through quantum mechanical techniques. The advantage here is that they can adapt to new chemical classes and do not necessarily need prior examples within the algorithm. In each case, and to differing degrees, estimates can be complicated by conformational flexibility, solvent handling, conjugated systems and a lack of relevant examples. The needs of the pharmaceutical industry are challenging as they regularly explore novel structural scaffolds to enter new patent territory. If the software requires prior examples of a functional group or scaffold then accuracy may be compromised. For the purposes of characterizing a database, speed of calculation is a priority and may take precedence over accuracy. There are many computational hurdles yet to be tackled to provide a chemist friendly, fast and accurate system of estimating p*K*_a_ values within large databases (100,000’s compounds). Among the considerations are problems such as conformational flexibility, internal hydrogen bonding, solvent effects and multiprotic influences^7^. Fortunately, several groups are working on better prediction methods and this will ultimately influence how we undertake research for new medicines.

## Conclusion

This study has begun to explore the overall composition of drugs with regard to the proportion of those compounds containing an ionizable group. Within the WHO essential medicines list 65.1% of compounds had an ionizable group with a p*K*_a_ in the range 2–12 and this number rises to 77.5% when non drug-like compounds are removed. Other estimates give this number as anywhere between 62.9%^29^,^30^ and 95%^27^. It is certainly clear that this figure is influenced by the collection being studied and how ‘ionizable’ is defined, and will be the subject of future research from our laboratories.

Analysis of Williams collection of drugs^33^ has led to a description of the relative proportions of compounds containing acidic and basic functionality. More importantly, the distribution of p*K*_a_ values has been outlined in detail for the first time. Two clear findings emerged upon examination of the distributions particularly when a distinction was made between CNS and non-CNS drugs. Firstly, acid p*K*_a_ values for CNS drugs rarely fell below 6.0 and secondly, base p*K*_a_ values for CNS drugs were not observed above a value of 10.5. From an ionization viewpoint these observations are entirely reasonable when considering the nature of the BBB and the passage of charged substances across membranes. As such, these observations consolidate current wisdom in the area and open the way for larger collections to be compared to these distributions.

Without doubt p*K*_a_ is of paramount importance to the overall characteristics of a drug and has considerable influence on biopharmaceutical properties. Current trends indicate that future research is placing an increased focus on p*K*_a_ with the advent of high throughput measurement techniques and improvements to computational prediction software^7^. By taking p*K*_a_ into account allows the researcher to begin ADME profiling early in the discovery process. Moreover, with large collections of compounds such as corporate databases, third party supplier offerings and virtual sets of compounds (e.g. virtual combinatorial libraries), the researcher can examine both the proportion of ionizable compounds and with prediction methods can start to look at p*K*_a_ distributions. If these differ largely from the observations outlined in the current study then it allows the opportunity to amend synthetic directions or screening compound selections.

The drive to consider the physicochemical properties of drugs to understand biopharmaceutical characteristics began many years ago (e.g.^10^). This has fundamentally changed how discovery work is undertaken and was oriented to improving the efficiency and productivity of pharmaceutical companies. Likewise, the need to explore p*K*_a_ will begin to influence how we work. The findings presented here go some way to understanding the distribution of p*K*_a_ values and further guidelines will evolve as larger datasets are analyzed.

## Supplementary Material

The Williams^33^ dataset has been provided as supplementary material.

## Figures and Tables

**Table 1.** A list of the number of acids and bases in the Williams dataset^33^ and associated subsets.

	**No acid**	**1 acid**	**2 acids**	**3 acids**
*Entire dataset*				
No base	0	142	22	3
1 base	264	65	16	4
2 bases	61	1	0	0
3 bases	4	0	0	0
*CNS subset*				
No base	0	27	2	0
1 base	108	13	0	1
2 bases	23	0	0	0
3 bases	0	0	0	0
*Non-CNS subset*				
No base	0	115	20	3
1 base	156	52	16	3
2 bases	38	1	0	0
3 bases	4	0	0	0

**Table 2.** Percentage of acid and base containing compounds in the Williams^33^ and WDI datasets^28^.

**List**	**1 acid**	**1 base**	**2 acids**	**2 bases**	**1 acid + 1 base**	**Others**
*Entire dataset*	24.4	45.4	3.8	10.5	11.2	4.8
*CNS subset*	15.5	62.1	1.1	13.2	7.5	0.6
*Non-CNS subset*	28.2	38.2	4.9	9.3	12.7	6.6
WDI*	11.6	42.9	3.0	24.6	7.5	10.4

* Data taken from Comer and Tam^29^,^30^
